# Invasive predator diet plasticity has implications for native fish conservation and invasive species suppression

**DOI:** 10.1371/journal.pone.0279099

**Published:** 2023-02-24

**Authors:** Hayley C. Glassic, Christopher S. Guy, Lusha M. Tronstad, Dominique R. Lujan, Michelle A. Briggs, Lindsey K. Albertson, Todd M. Koel

**Affiliations:** 1 Montana Cooperative Fishery Research Unit, Department of Ecology, Montana State University, Bozeman, Montana, United States of America; 2 Department of Ecology, U.S. Geological Survey, Montana Cooperative Fishery Research Unit, Montana State University, Bozeman, Montana, United States of America; 3 Wyoming Natural Diversity Database, University of Wyoming, Laramie, Wyoming, United States of America; 4 Department of Zoology and Physiology, University of Wyoming, Laramie, Wyoming, United States of America; 5 Department of Ecology, Montana State University, Bozeman, Montana, United States of America; 6 U.S. National Park Service, Yellowstone Center for Resources, Native Fish Conservation Program, Yellowstone National Park, Wyoming, United States of America; Universita del Salento, ITALY

## Abstract

Diet plasticity is a common behavior exhibited by piscivores to sustain predator biomass when preferred prey biomass is reduced. Invasive piscivore diet plasticity could complicate suppression success; thus, understanding invasive predator consumption is insightful to meeting conservation targets. Here, we determine if diet plasticity exists in an invasive apex piscivore and whether plasticity could influence native species recovery benchmarks and invasive species suppression goals. We compared diet and stable isotope signatures of invasive lake trout and native Yellowstone cutthroat trout (cutthroat trout) from Yellowstone Lake, Wyoming, U.S.A. as a function of no, low-, moderate-, and high-lake trout density states. Lake trout exhibited plasticity in relation to their density; consumption of cutthroat trout decreased 5-fold (diet proportion from 0.89 to 0.18) from low- to high-density state. During the high-density state, lake trout switched to amphipods, which were also consumed by cutthroat trout, resulting in high diet overlap (Schoener’s index value, D = 0.68) between the species. As suppression reduced lake trout densities (moderate-density state), more cutthroat trout were consumed (proportion of cutthroat trout = 0.42), and diet overlap was released between the species (D = 0.30). A shift in lake trout δ^13^C signatures from the high- to the moderate-density state also corroborated increased consumption of cutthroat trout and lake trout diet plasticity. Observed declines in lake trout are not commensurate with expected cutthroat trout recovery due to lake trout diet plasticity. The abundance of the native species in need of conservation may take longer to recover due to the diet plasticity of the invasive species. The changes observed in diet, diet overlap, and isotopes associated with predator suppression provides more insight into conservation and suppression dynamics than using predator and prey biomass alone. By understanding these dynamics, we can better prepare conservation programs for potential feedbacks caused by invasive species suppression.

## Introduction

Invasive species are the second greatest threat to biodiversity loss in North America behind habitat loss [1] because invasive species can induce ecosystem collapse [2, 3]. Specifically, invasive species are a leading threat to aquatic ecosystems [4, 5] and influence native fishes through predation, competition, and introgressive hybridization [6]. Apex piscivores are some of the most harmful invasive species [7, 8] and prey species are usually highly vulnerable to the specific predation behaviors of the newly introduced predator [7]. Characteristics of invasive species, such as maximum body size, physiological tolerance [9], fecundity [9, 10], and diet plasticity [11, 12] aid in their establishment or expansion in a novel ecosystem. Moreover, invasive piscivores can compete with native prey species during juvenile stages because most piscivorous fishes exhibit ontogenetic diet shifts [13]. Consequently, food-web structure is often altered when fishes invade [14–18], resulting in trophic cascades within and across aquatic-terrestrial ecosystem boundaries [19–22].

Much of the concern regarding apex invasive predators is focused on their ability to reduce native species through predation [23–27]. Diet plasticity is common in apex predators, whether native or invasive. When preferred prey abundance decreases, apex predators maintain fitness, density, or biomass by shifting their diet [11, 12, 28, 29], exhibiting diet plasticity. Prey switching can complicate the suppression of invasive apex predators on native species when declines in the invasive predator are not commensurate with the recovery of preferred prey species. Conserving native species and maintaining ecosystem function are the central tenant for many invasive species suppression programs [30–34]. Understanding the effect of prey switching by an apex predator is important for establishing realistic suppression targets and conservation benchmarks in invaded ecosystems.

One of the largest apex invasive species suppression programs in the world occurs in Yellowstone Lake, Yellowstone National Park, Wyoming, USA where invasive lake trout (*Salvelinus namaycush*) are suppressed to conserve native Yellowstone cutthroat trout (*Oncorhynchus clarkii bouvieri*, hereafter referred to as cutthroat trout) [31]. Lake trout are an apex predator native to Alaska, northern Canada, Laurentian Great Lakes, and parts of New England [35], but have been introduced to 15 countries and extensively throughout the western United States [36]. Predatory demand of introduced lake trout caused declines in native fish populations [37–40] and altered ecosystem structure and function [19, 21, 40], sometimes even before lake trout reach high densities [41]. Lake trout not only degrade native food webs, but also cause major economic loss by altering fisheries and warranting expensive suppression programs or hatchery-based native recovery programs [31, 39, 42].

**Fig 1:**
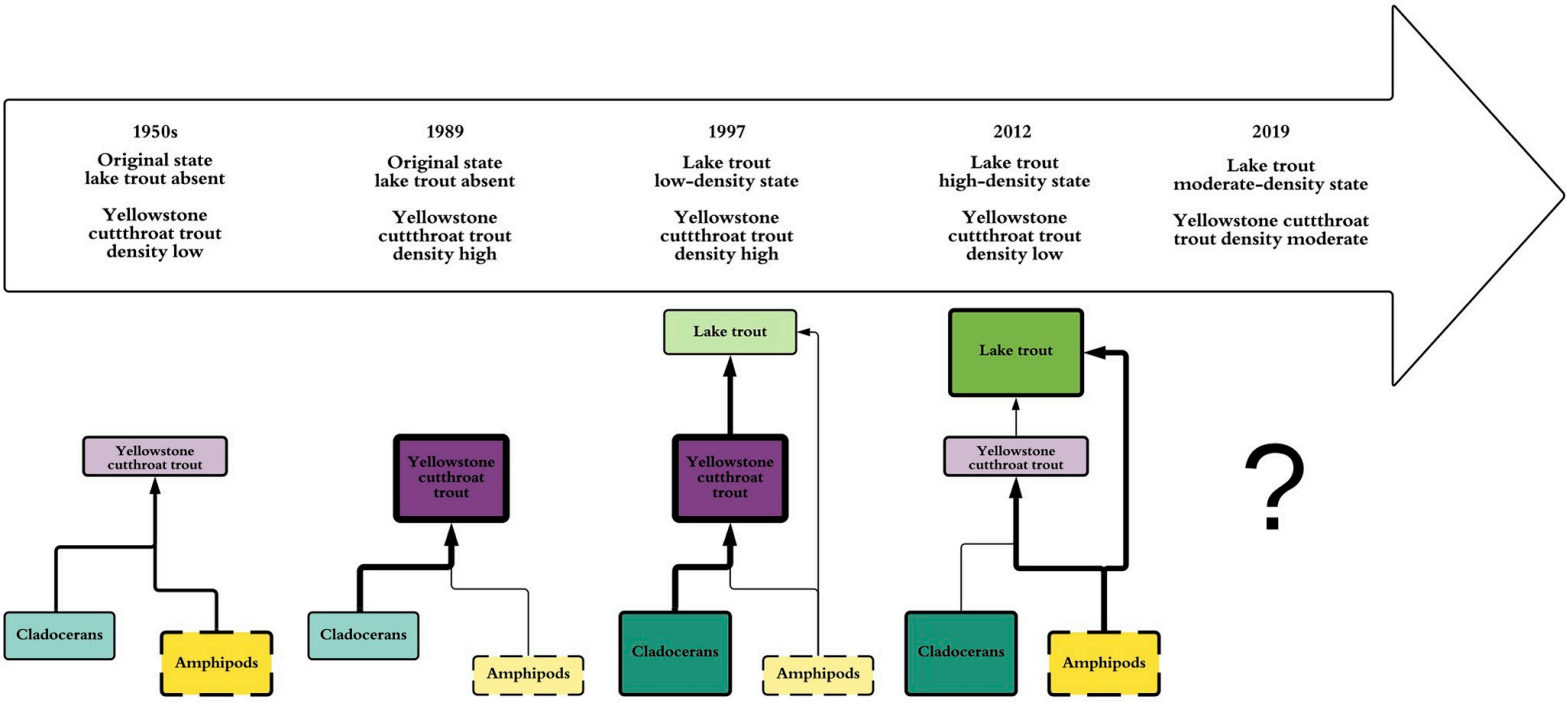
Timeline of simplified trout diets. Historical representations of Yellowstone cutthroat trout and >300 mm lake trout diets before lake trout invasion, and during two stages of lake trout density, with the current diet composition unknown during a moderate-density state of lake trout. Size, color intensity, and thickness of solid-lined boxes represents relative densities of organisms, thickness of lines represents relative contributions of organisms to predator diet, and size, color intensity, and thickness of dashed boxes represents inferred density of organisms based on previous research. Diet data for the 1950s are from Benson [44], data for 1989 are from Jones et al. [46], data for 1997 are from Ruzycki et al. [54], and data for 2012 are from Syslo et al. [53]. Relative abundances of lake trout are from Syslo et al. [55]. Lake trout absent is a state with no detectable density, low-density state is ~80,000 >300 mm lake trout, high density is ~450,000 >300 mm lake trout, and moderate-density state is ~300,000 >300 mm lake trout. Relative abundances of Yellowstone cutthroat trout are from Koel et al. [40]. Relative abundances of cladocerans are from Tronstad et al. [21], and relative abundances of amphipods are inferred from Wilmot et al. [52].

In Yellowstone Lake, invasive lake trout are predators of the native cutthroat trout population, which represents the largest population of nonhybridized cutthroat trout in existence [43]. Several diet studies [44–47] were conducted on cutthroat trout before lake trout invaded and found that more cutthroat trout consumed amphipods when cutthroat trout density was low in the 1950s (Fig 1). Conversely, when cutthroat trout density was high in 1989, more zooplankton were consumed [45, 47, 48] (Fig 1). Increases in lake trout and declines in cutthroat trout due to lake trout predation caused a trophic cascade within the lake [21] that extended to tributaries [48] and the terrestrial ecosystem [40, 49–51]. The lake trout invasion also induced spatial variation in benthic invertebrate biomass [52]. The National Park Service (NPS) initiated a lake trout suppression program in 1995 with the purpose of reducing lake trout abundance [53] to decrease predation on cutthroat trout [54] and prevent ecosystem collapse. With the introduction and expansion of lake trout in Yellowstone Lake, trophic cascade [21], and spatial variation in benthic invertebrates [52], studies focusing on the diets of lake trout and cutthroat trout [53, 54] were conducted to identify diet composition and describe potential diet shifts. During lake trout expansion in 1997, larger (>300 mm total length) lake trout consumed cutthroat trout in high proportion [54] (Fig 1), while zooplankton dominated cutthroat trout diet (Fig 1). Syslo et al. [53] described lake trout and cutthroat trout consuming primarily amphipods in 2012 and having high dietary overlap (Fig 1). This research showed a shift in invasive predator and native prey consumption during the period of highest lake trout density [55] and lowest cutthroat trout density [31].

Examining the plasticity in diet composition, diet overlap, and trophic position of invasive and native fishes in altered ecosystems is insightful for determining the effects of introduced species and establishing recovery benchmarks of native species. Diet studies typically focus on the effects of invasive piscivores on native fishes or possible trophic overlap during initial invasion [56, 57]; however, quantifying long-term changes in diet composition and trophic position is rare [53]. Additionally, the description of diet composition for invasive fishes during low introductory density, peak density during expansion, and reduced density due to suppression has not yet been studied. Thus, we designed our study to have two main components. First, we describe the status of cutthroat trout and lake trout diets and stable isotope signatures using similar methodology to historical studies for equivalent comparison. Second, we answer the following questions: 1) do invasive predator and native prey diets vary as a function of predator and prey density; 2) does strength of diet similarity (i.e., overlap) between the invasive predator and native prey vary as a function of predator and prey density; 3) if plasticity exists in predator diets, what are the effects of plasticity on realistically attaining management benchmarks for the species requiring conservation? These questions are within the context of the conservation of native species via invasive species suppression. Understanding the predator-prey dynamics in the Yellowstone Lake ecosystem will better inform invasive species suppression efforts and time required to achieve conservation benchmarks for native species.

## Methods

### Study site

**Fig 2:**
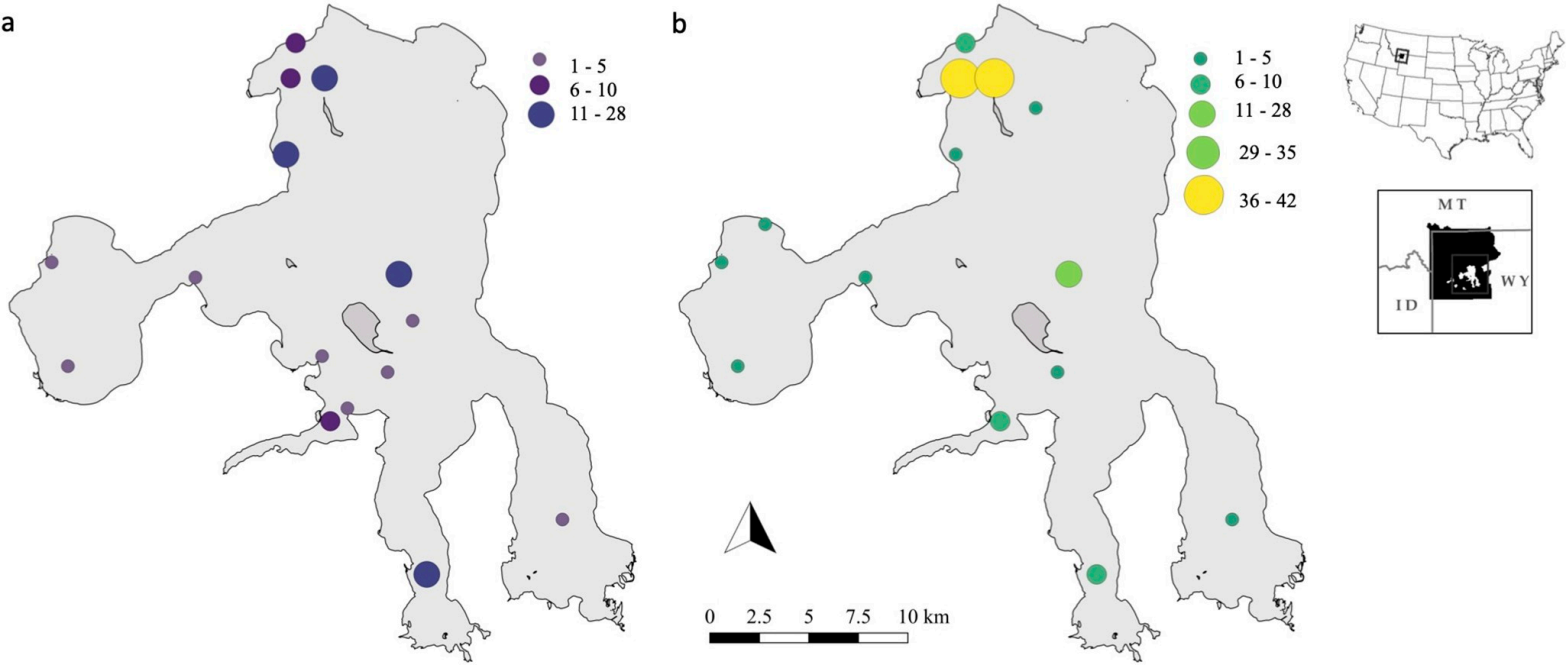
Distribution of samples collected from Yellowstone Lake. Map of Yellowstone Lake, Yellowstone National Park, Wyoming, USA showing the number and location of stable isotope samples and corresponding diet samples for Yellowstone cutthroat trout (a, purple hues) and lake trout (b, green hues) collected in 2018 and 2019. Base map sources can be accessed using the following: Yellowstone Lake border (https://pubs.usgs.gov/sim/2007/2973/); United States of America and individual state borders (https://www.sciencebase.gov/catalog/item/52c78623e4b060b9ebca5be5); Yellowstone National Park border (https://www.sciencebase.gov/catalog/item/4ffb3aebe4b0c15d5ce9fc0b).

Yellowstone Lake is a large, oligo-mesotrophic lake [58] located in Yellowstone National Park, Wyoming, USA (Fig 2), and is the largest lake above 2,000 m elevation in North America with a surface area of 34,020 ha, a mean depth of 48 m, and a maximum depth of 133 m [59]. The lake is typically ice covered from late-December until late May or early June. Water temperatures fluctuate between 9°C and 18°C in the summer and a thermocline develops during stratification from July through mid-September at about 15 m [31]. Diatoms dominate the phytoplankton assemblage [21, 44]. The zooplankton community is primarily composed of rotifers *Conochilus unicornis*, copepods *Diacyclops bicuspidatus thomasi*, *Leptodiaptomus ashlandi*, and *Hesperodiaptomus shoshone* [21], and cladocerans *Daphnia* spp. [44]. Amphipods *Hyalella azteca* and *Gammarus lacustris* are the most common benthic macroinvertebrate [44, 52]. Native fishes in Yellowstone Lake include Yellowstone cutthroat trout and longnose dace *Rhinichthys cataractae*. In addition to invasive lake trout, other nonnative species unintentionally introduced as baitfish include longnose sucker *Catostomus*, redside shiner *Richardsonius balteatus*, and lake chub *Couesius plumbeus* [60]. The other nonnative fishes were rarely studied, but may have influenced the ecosystem by consuming plankton and macroinvertebrates [44, 61–63]. No evidence exists to suggest that these fishes negatively influenced the native cutthroat trout [60–62, 64].

### Field sampling and laboratory processing

Fishes were sampled throughout Yellowstone Lake (Fig 2) during the ice-free season in 2018 and 2019 using gillnetting methods established by the NPS (see [31] for specifics on gillnetting placement and design). Diet samples of cutthroat trout and lake trout were collected by season: pre-stratification (before 1 August), stratification (1 August– 20 September), and post-stratification (after 20 September)—identical to Syslo et al. [53]. We sampled multiple individuals of each species in 50-mm total length classes starting at 100 mm during each season to account for ontogenetic diet shifts. Stomachs from cutthroat trout and lake trout that inadvertently died from gillnetting events were extracted and preserved in 70% ethanol. We pooled diet data among stratification seasons for subsequent analyses to more accurately complement stable isotope analysis from Syslo et al. [53], where stable isotope samples were not collected based on stratification season.

Fish tissue samples (~10 g of dorsal muscle tissue) were collected for stable isotope analysis. Methods for tissue collection, storage, and preparation were consistent with Syslo et al. [53]. Samples were analyzed at the University of Wyoming Stable Isotope Facility using an elemental analyzer (Thermo Finnigan Delta Plus XP, Costech 4010 and Carlo Erba 1110 Elemental Analyzer, Costech Zero Blank Autosampler, and Finnigan Conflo III Interface). Liver was used as the quality assurance material. The quality assurance of the isotope analysis is based on the standard uncertainty of the known value of the quality control reference materials analyzed during the analytical run. The standard uncertainty (1-sigma) is calculated from multiple analyses of the quality control reference materials. Stable isotope ratios were calculated using standard procedures outlined in Vander Zanden et al. [65] and Hershey et al. [66].

Stomach contents were analyzed for proportion of diet by wet mass [67], prey items were identified and separated by taxon, and the blotted wet weights were measured using the same methods as Ruzycki et al. [54] and Syslo et al. [53], thus studies were directly comparable. Invertebrates were identified to order or family and fishes were identified to species. Taxonomic identification categories were selected to match methodology used by Jones et al. [46], Ruzycki et al. [54], and Syslo et al. [53] and were defined as: cladocerans, copepods, amphipods, leeches, chironomids, insects (which included Ephemeroptera, Trichoptera, Plecoptera, and non-chironomid Diptera), mollusks, cutthroat trout, and unidentified fish. All field and laboratory sampling was conducted under Yellowstone National Park permit 8048. This study was performed under the auspices of Institutional Animal Care and Use Protocol 2018–72 at Montana State University.

### Analyses

We did not include cutthroat trout diet data from Benson [44] in statistical comparisons because we only had published, summarized data from the study. Statistical comparisons included in our study were conducted using data from Jones et al. [46] (lake trout absent), Ruzycki et al. [54] (low-density state), Syslo et al. [53] (high-density state), and this study (moderate-density state). All analyses were conducted using R [68] (version 4.1.3). We used diet and stable isotopes to represent short- and long-term integrations of diet. We wanted to explore potential change in diet by comparing raw diet, represented by diet proportion and stable isotope signature, and diet similarity, represented by overlap index and isotopic overlap.

### Diet

#### Proportion

We bootstrapped 95% credible intervals for the mean diet proportion for each diet item using methods from Olson [69]. We first described the diet proportions for cutthroat trout and lake trout during the moderate-density state and compared length classes within species. We made diet proportion comparisons within length class among density states to determine whether diet changed with predator density. A difference in diet proportion within length class and among states would be supported if 95% credible intervals did not overlap. For cutthroat trout, change among density states focused on differences in proportion of amphipods and cladocerans in diet, as these items were of highest proportion in previous studies [44, 46, 53]. For lake trout, change among density states focused on differences in proportion of amphipods and cutthroat trout in the diet, as these diet items were of highest proportion in previous studies [53, 54].

#### Stable isotope signature

We compared δ^15^N and δ^13^C by length group and species to determine whether stable isotope signature or trophic position: 1) was different between species within moderate-density state and 2) varied between high-density and moderate-density states, as only those states had stable isotope data available. A difference in isotopic signature would exist when comparing species, length classes, or density states if 95% confidence intervals around the mean signature did not overlap.

### Diet similarity

#### Overlap index

We wanted to address the similarity between predator and prey diets in relation to density state as similarity can contextualize potential for competition. First, we compared diet similarity between cutthroat trout and lake trout for the moderate-density state only. We then compared diet similarity for high- and moderate-density states because diet data for comparisons to low-density state were not available for cutthroat trout, and predator-prey comparisons could not be made during absent states.

Diet overlap between lake trout and cutthroat trout by length group was calculated using Schoener’s index of niche overlap:

$$\text{D}=1–0.5\;(\sum_{i=1}^{n}\left|p_{ij}-p_{ik}\right|),$$

where *p*_*ij*_ is the contributed proportion of prey type *i* to the diet for species *j* and *p*_*ik*_ is the contributed proportion of prey type *i* to the diet for species *k* [70]. Values of D ≥ 0.60 indicated a high degree of diet overlap [71].

#### Isotopic overlap

We used SIBER package in R [72] (version 2.1.4) to create 40% Bayesian standard ellipses and to calculate percent of ellipse overlap. In addition to the Schoener’s index of diet overlap, isotopic ellipse overlap can represent potential diet overlap between species in Yellowstone Lake. We used the same criteria as Schoener’s diet index [71], where isotopic overlap ≥ 60% was considered to indicate a high degree of isotopic overlap [73, 74]. We compared overlap between species within the moderate-density state and then made comparisons between the high- and moderate-density states.

## Results

### Comparisons within lake trout moderate-density state

#### Diet proportion

**Fig 3:**
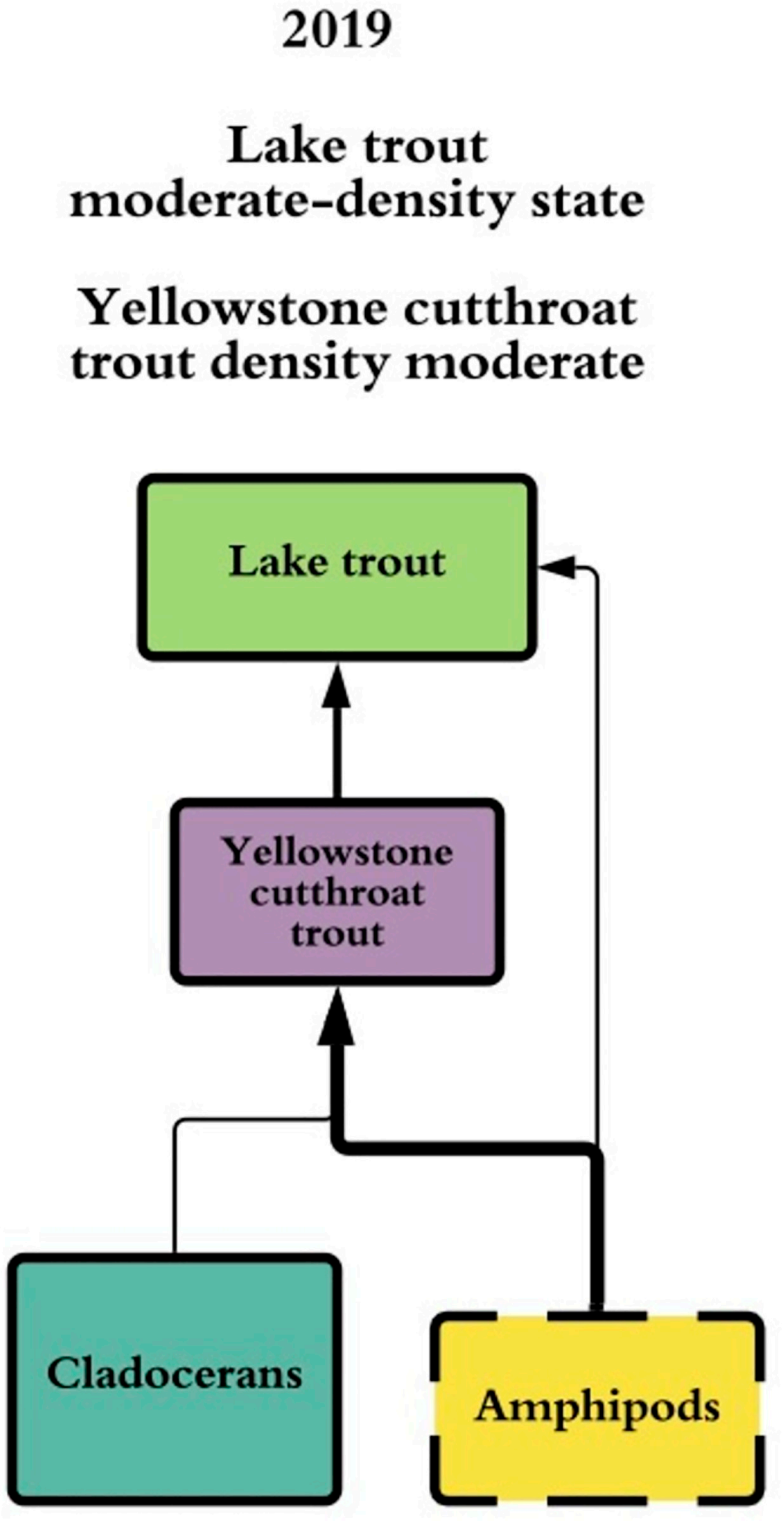
Contemporary diet. Simplified representation of Yellowstone cutthroat trout and >300 mm lake trout diets during the moderate-density state (~300,000 >300 mm lake trout; estimate of lake trout density from Syslo et al. [55]). Size and thickness of solid-lined boxes represents relative densities of organisms, thickness of lines represent relative contributions of organisms to predator diet, size and thickness of dashed boxes represents inferred density of organisms based on previous research.

**Fig 4:**
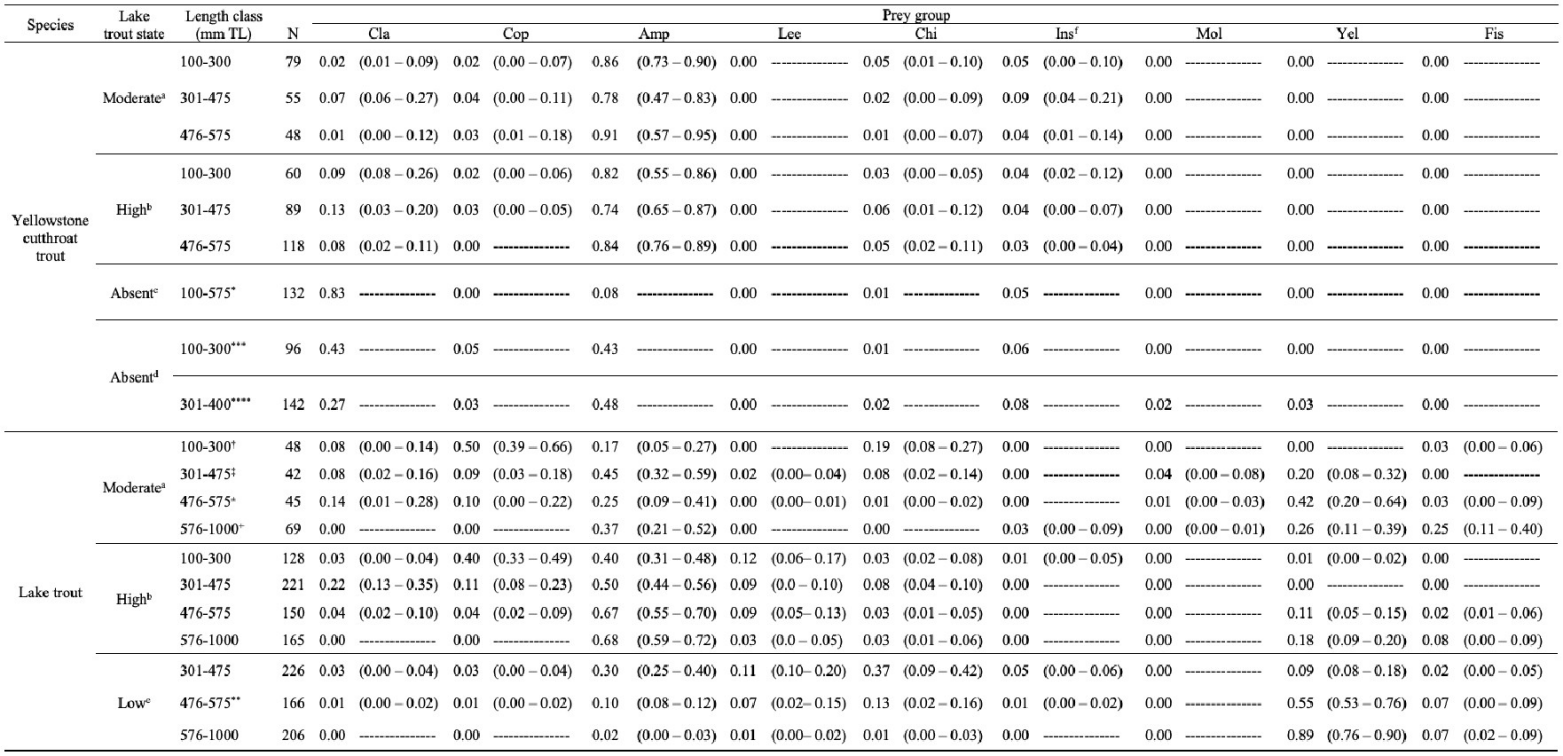
Comparisons of diet proportions. Diet composition (proportion by weight) for Yellowstone cutthroat trout and lake trout in Yellowstone Lake (Cla = cladocerans; Cop = copepods; Amp = amphipods; Lee = leeches; Chi = chironomids; Ins = insects; Mol = mollusks; Yel = Yellowstone cutthroat trout; Fis = unidentified fish). Bootstrapped 95% credible intervals were drawn from a distribution of 1000 samples. Relative abundances of lake trout are from Syslo et al. [55], where absent is a state with no detectable density or complete absence of lake trout (1989), low-density state is ~80,000 >300 mm lake trout (1997), high-density state is ~450,000 >300 mm lake trout (2012), and moderate-density state is ~300,000 >300 mm lake trout (2019). No Yellowstone cutthroat trout diets were analyzed during the lake trout low-density state. ^a^Current study. ^b^Syslo et al. [53] averages among stratification seasons. ^c^Jones et al. [46]. ^d^Benson [44]. ^e^Ruzycki et al. [54]. ^f^Includes Ephemeroptera, Tricoptera, Plecoptera, and non-chironomid dipterans. *Not included in table are diet items and proportions: 0.01 Leucisids, 0.02 organic matter. ^†^Not included in table are diet items and proportions: 0.01 diatoms, 0.02 detritus. ^‡^Not included in table are diet items and proportions: 0.03 diatoms, 0.01 detritus. ^±^Not included in table are diet items and proportions: 0.01 diatoms, 0.03 detritus. ^+^Not included in table are diet items and proportions: 0.06 diatoms, 0.03 detritus. **Not included in table are diet items and proportions: 0.04 lake trout eggs, 0.01 sucker. ***Not included in table are diet items and proportions: 0.01 wasps and beetles, 0.01 water mites. ****Not included in table are diet items and proportions: 0.03 wasps and beetles, 0.02 water mites, 0.02 gastropods.

The main diet of cutthroat trout (n = 182; empty stomachs not included in analysis) during the moderate-density state was amphipods (Figs 3 and 4). Amphipods composed >0.75 of the diet by proportional weight in all total length classes in the moderate-density state (Fig 4), followed by insects. The third most consumed diet items by weight were chironomids for the 100–300 mm length class, cladocerans for the 301–475 mm length class, and copepods for the 476–575 mm length class (Fig 4). However, 95% credible intervals (CI) overlapped for many of the diet item proportions within and among length class.

We analyzed the stomach contents of 204 lake trout (empty stomachs not included in analysis) from the moderate-density state. The main diet items for lake trout >300 mm were amphipods and cutthroat trout (Figs 3 and 4). Copepods composed the largest proportion of diet by weight for lake trout in the 100–300 mm length class with chironomids as the secondary diet item and amphipods as the tertiary diet item (Fig 4). Amphipods composed 0.45 (0.32–0.59 CI) and cutthroat trout composed 0.20 (0.08–0.32 CI) of diet weight for 301–475 mm lake trout—indicating that piscivory by lake trout on cutthroat trout begins when lake trout are approximately >300 mm in length (Fig 4). Larger lake trout (476–575 mm length) were primarily piscivorous; 0.42 (0.20–0.64 CI) contents by weight were confirmed cutthroat trout. Lake trout 576–1000 mm length class consumed 0.51 proportion of fish of which 0.26 (0.11–0.39 CI) were confirmed cutthroat trout and 0.25 (0.11–0.40 CI) were unidentified fish (Fig 4). Four fully intact cutthroat trout were recovered from lake trout diets with an average total length of 187.8 mm (54.7 standard deviation). Amphipods composed 0.25 (0.09–0.41 CI) of diet weight for the 476–575 mm length class and 0.37 (0.21–0.52) of diet weight for the 576–1000 mm length class of lake trout.

#### Overlap index

**Fig 5:**
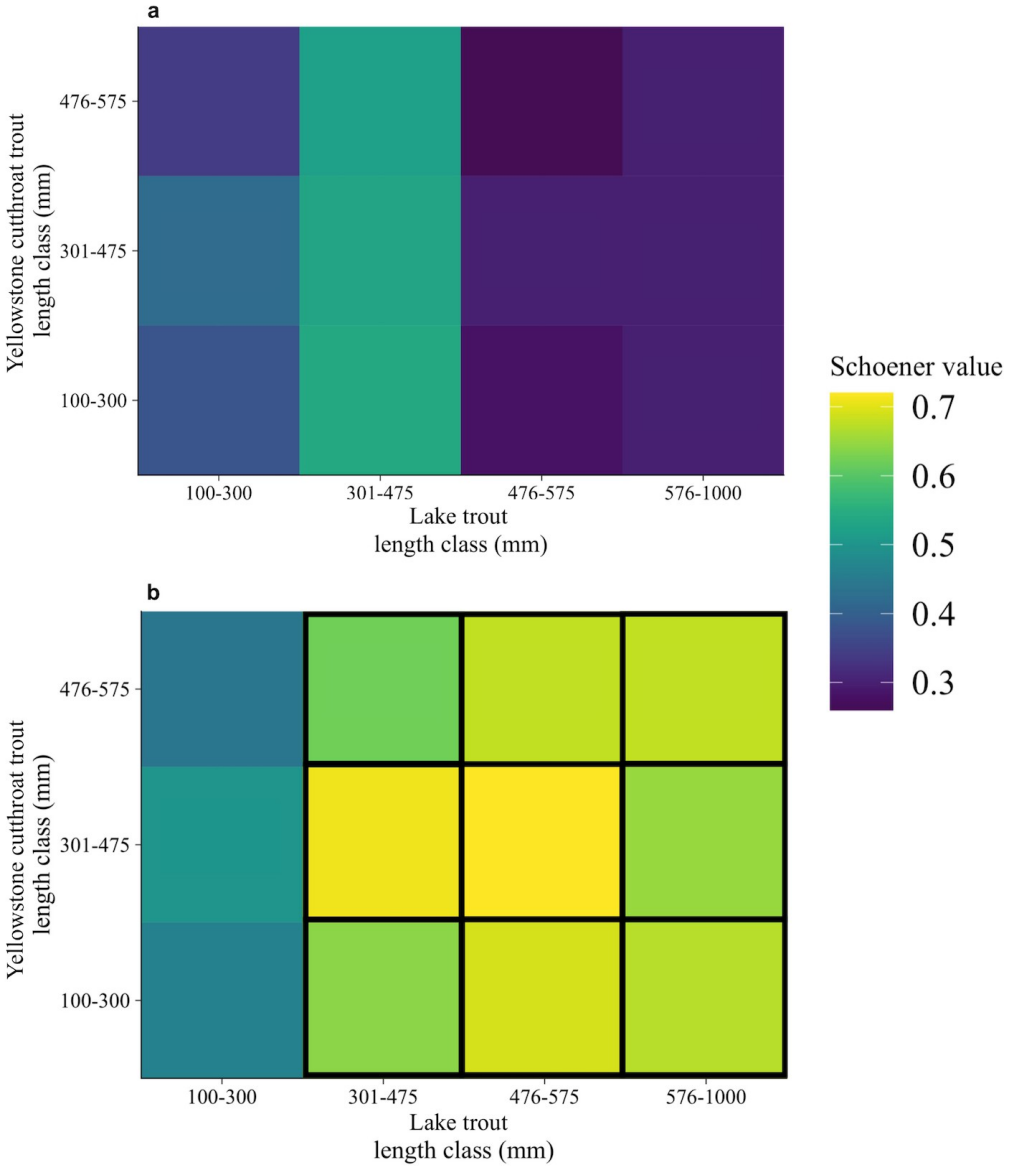
Comparison of diet overlap between two lake trout density states. Schoener’s index of diet overlap values for lake trout moderate-density state (this study) (a) and a lake trout high-density state (Syslo et al. [53]) (b). Relative abundances of lake trout are from Syslo et al. [55], where high density is ~450,000 >300 mm lake trout, and moderate-density state is ~300,000 >300 mm lake trout. Schoener values >0.6 indicate a high degree of overlap (Wallace [67]), represented by boxes with black outlines.

During the moderate-density state, Schoener’s index of diet overlap was <0.6 for all combinations of species and length class (Fig 5a). Diet overlap was lowest between 476–575 mm cutthroat trout and lake trout (Fig 5a) and was highest among all cutthroat trout length classes and 301–475 mm lake trout (Fig 5a).

#### Stable isotope signature

**Fig 6:**
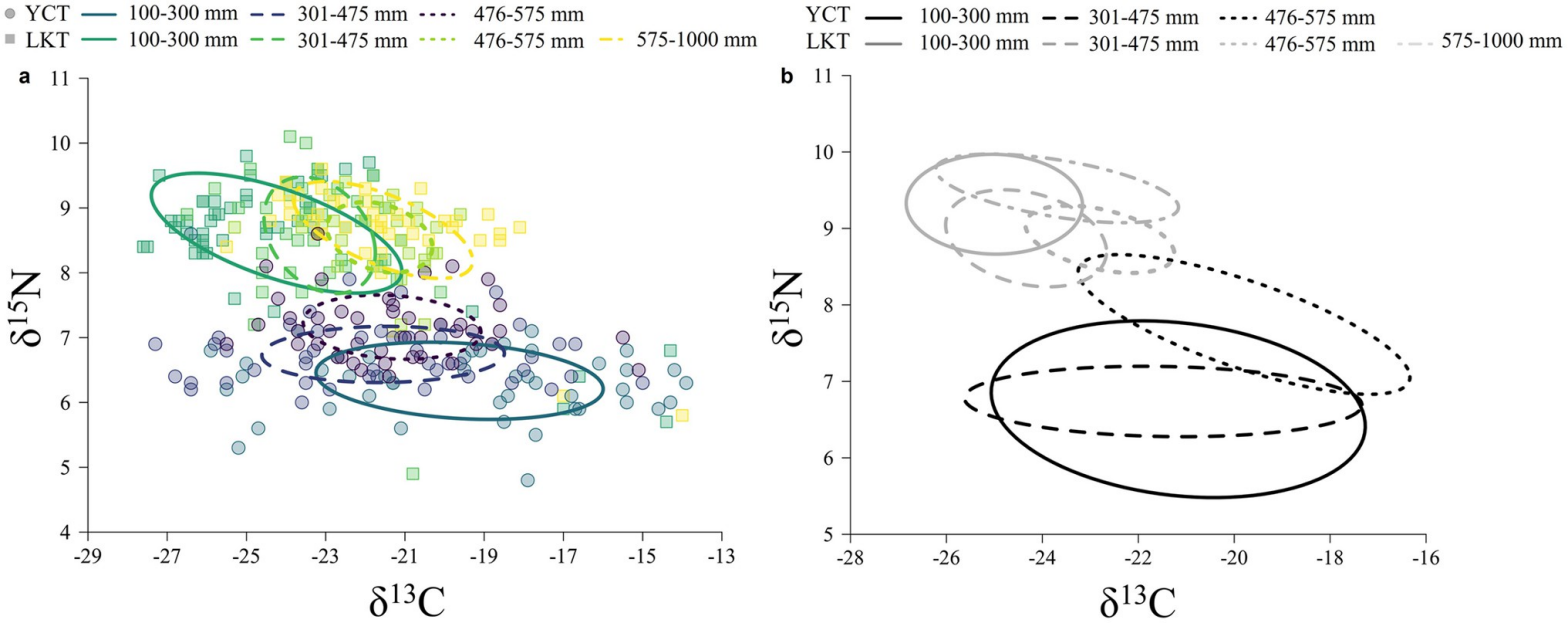
Comparison of stable isotope signatures and ellipse areas between native and invasive trout. Individual stable isotope values (δ15N, δ13C) and standard Bayesian ellipse areas for Yellowstone cutthroat trout (YCT) length classes (circles; purple hues) and lake trout (LKT) length-classes (squares; green hues) sampled from Yellowstone Lake, 2018–2019, during moderate density state (a) and standard Bayesian ellipse areas for high-density state Yellowstone cutthroat trout (YCT) length classes (black) and lake trout (LKT) length-classes (grey) from Syslo et al. [53] (b). Note that the scales of x-axes on the panels are different.

Cutthroat trout (n = 137) and lake trout (n = 161) occupy different trophic space for the moderate-density state in Yellowstone Lake (Table 1; Fig 6a). Values of δ^13^C varied from –27.40‰ to –13.90‰ for cutthroat trout (Fig 6a) and from –27.60‰ to –14.00‰ for lake trout (Fig 6a). Values of δ^15^N varied from 4.80‰ to 8.60‰ for cutthroat trout (Fig 6a) and from 4.90‰ to 10.10‰ for lake trout (Fig 6a). All length classes of lake trout were enriched in δ^15^N relative to all cutthroat trout length classes. Cutthroat trout had on average higher values of δ^13^C compared to lake trout (Fig 6a).

**Table 1:** Comparison of stable isotope signatures for native and invasive trout between lake trout density states. Sample sizes (N) and mean δ13C and δ15N (95% confidence intervals in parentheses) and median posterior distribution value of standard Bayesian ellipse areas (SEA_B_) by lake trout density state (lake trout state) and length class for lake trout and Yellowstone cutthroat trout. Amphipods included to demonstrate basal isotope values. Lake trout state densities are from Syslo et al. [55].

Species	Lake trout state (abundance of >300 mm)	Length class (mm TL)	N	δ^13^C (‰)	δ^15^N (‰)	SEA_B_
Yellowstone cutthroat trout	Moderate^a^ (~300,000)	100–300	45	-19.62 (-20.66, -18.58)	6.33 (6.15, 6.51)	6.55
		301–475	48	-21.55 (-22.41, -20.69)	6.73 (6.61, 6.85)	4.10
		476–575	44	-21.32 (-21.99, -20.65)	7.16 (7.03, 7.30)	3.42
	High^b^ (~450,000)	100–300	12	-21.17 (-23.34, -18.99)	6.64 (5.99, 7.29)	13.9
		301–475	17	-21.48 (-23.42, -19.54)	6.73 (6.51, 6.95)	5.99
		476–575	16	-19.80 (-21.49, -18.11)	7.74 (7.29, 8.19)	7.01
Lake trout	Moderate^a^ (~300,000)	100–300	48	-24.24 (-25.12, -23.36)	8.61 (8.36, 8.87)	7.12
		301–475	42	-23.15 (-23.56, -22.74)	8.58 (8.31, 8.85)	3.72
		476–575	28	-21.63 (-22.12, -21.14)	8.54 (8.34, 8.74)	2.16
		576–1000	43	-21.55 (-22.22, -20.88)	8.66 (8.44, 8.88)	4.11
	High^b^ (~450,000)	100–300	25	-25.00 (-25.71, -24.29)	9.32 (9.07, 9.58)	3.75
		301–475	18	-24.34 (-25.10, -23.58)	8.87 (8.58, 9.16)	3.22
		476–575	10	-22.80 (-23.76, -21.84)	8.86 (8.59, 9.13)	1.99
		576–1000	17	-23.70 (-24.90, -22.50)	9.52 (9.30, 9.74)	3.03
Amphipods	Moderate^a^ (~300,000)	No length class	201	-12.98 (-13.53, -12.42)	2.25 (2.02, 2.48)	NA
	High^b^ (~450,000)	No length class	21	-15.26 (-17.71, -12.82)	3.90 (3.06, 4.75)	NA

^a^Current study

^b^Syslo et al. [53]

#### Isotopic overlap

Standard Bayesian ellipses did not overlap between species during the moderate-density state (Fig 6a). Overlap was present within species among length classes. Cutthroat trout overlap was <60% for all length class comparisons, with 100–300 mm and 301–475 mm length classes having the most overlap at 38.4% (Fig 6a). Lake trout overlap was high for most comparisons. Overlap was >60% for comparison between 475–575 mm and 576–1000 mm lake trout at 94.4% and was 83.7% for comparison between 100–300 mm and 301–475 mm lake trout (Fig 6a).

### Comparisons among density states

#### Diet proportion

Diets of cutthroat trout varied among lake trout density states with reliance on amphipods more common when cutthroat trout densities were low. Cutthroat trout shifted from an amphipod-rich diet when lake trout were absent and cutthroat density was low (0.48 amphipods), to a cladoceran-rich diet when lake trout were absent and cutthroat density was high (0.83 cladocerans), to an amphipod-rich diet during the lake trout high-density state (mean 0.79 among length classes), and the diet continued to be primarily amphipods in the lake trout moderate-density state (mean 0.85 among length classes; Fig 4).

Diets of lake trout varied among lake trout density states with piscivory more common at lower lake trout densities. Diet proportion of copepods in the 100–300 mm lake trout length class was 0.40 (0.33–0.49 CI) during the high-density state (2011–2013), and 0.50 (0.39–0.66 CI) during the moderate-density state (Fig 4). Lake trout in the 301–475 mm, 476–575 mm, and 576–100 mm length classes shifted from consuming primarily cutthroat trout during the low-density state to a diet of primarily amphipods during the high-density state and reverted to consuming cutthroat trout during the moderate-density state (Fig 4).

#### Overlap index

As lake trout density decreased from high to moderate, lake trout and cutthroat trout diet overlap was released. During the high-density state, D values were >0.60 for half of the comparisons between cutthroat trout and lake trout from varying length-classes (Fig 5b). Diet similarity during the high-density state was on average two times higher for comparisons among >300 mm lake trout and all cutthroat trout length classes (Fig 5a & 5b).

#### Stable isotope signature

Cutthroat trout trophic position did not change in response to lake trout density because 95% confidence intervals overlapped for all δ^15^N means, although a change in lake trout trophic position was observed. No evidence supported a difference in cutthroat trout δ^13^C and δ^15^N signatures between high- and moderate-density states because 95% confidence intervals overlapped for all δ^13^C and δ^15^N means (Table 1). However, evidence existed to support a difference in δ^13^C and δ^15^N for lake trout in some length classes between high- and moderate-density states because 95% confidence intervals did not overlap (Table 1). Data suggested more negative δ^13^C signatures for the high- than the moderate-density state for the 301–475 mm and 576–1000 mm lake trout. Higher δ^15^N signatures were observed for the high-density state than the moderate-density state for the 100–300 mm and 576–1000 mm lake trout (Table 1).

#### Isotopic overlap

**Fig 7:**
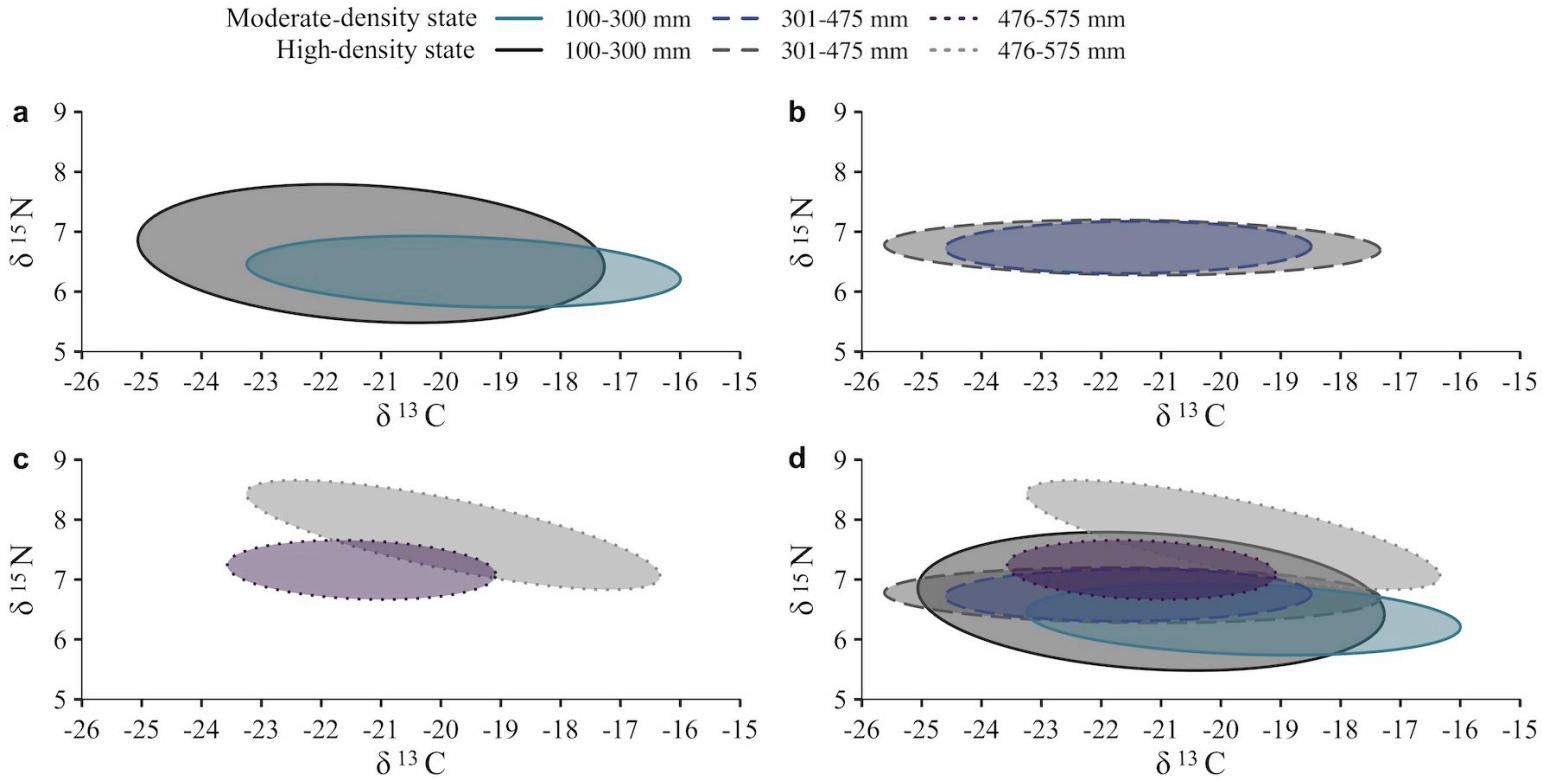
Stable isotope ellipse overlap for Yellowstone cutthroat trout in two lake trout density states. Standard Bayesian ellipse area 40% core distribution of stable isotope signatures for Yellowstone cutthroat trout from a lake trout moderate-density state (purple-blue hues) and a lake trout high-density state (Syslo et al. [53]; grey hues) among length classes: 100–300 mm (a), 301–475 mm (b), 476–575 mm (c), and all length classes (d). Relative abundances of lake trout are from Syslo et al. [55], where high density is ~450,000 >300 mm lake trout, and moderate-density state is ~300,000 >300 mm lake trout.

**Fig 8:**
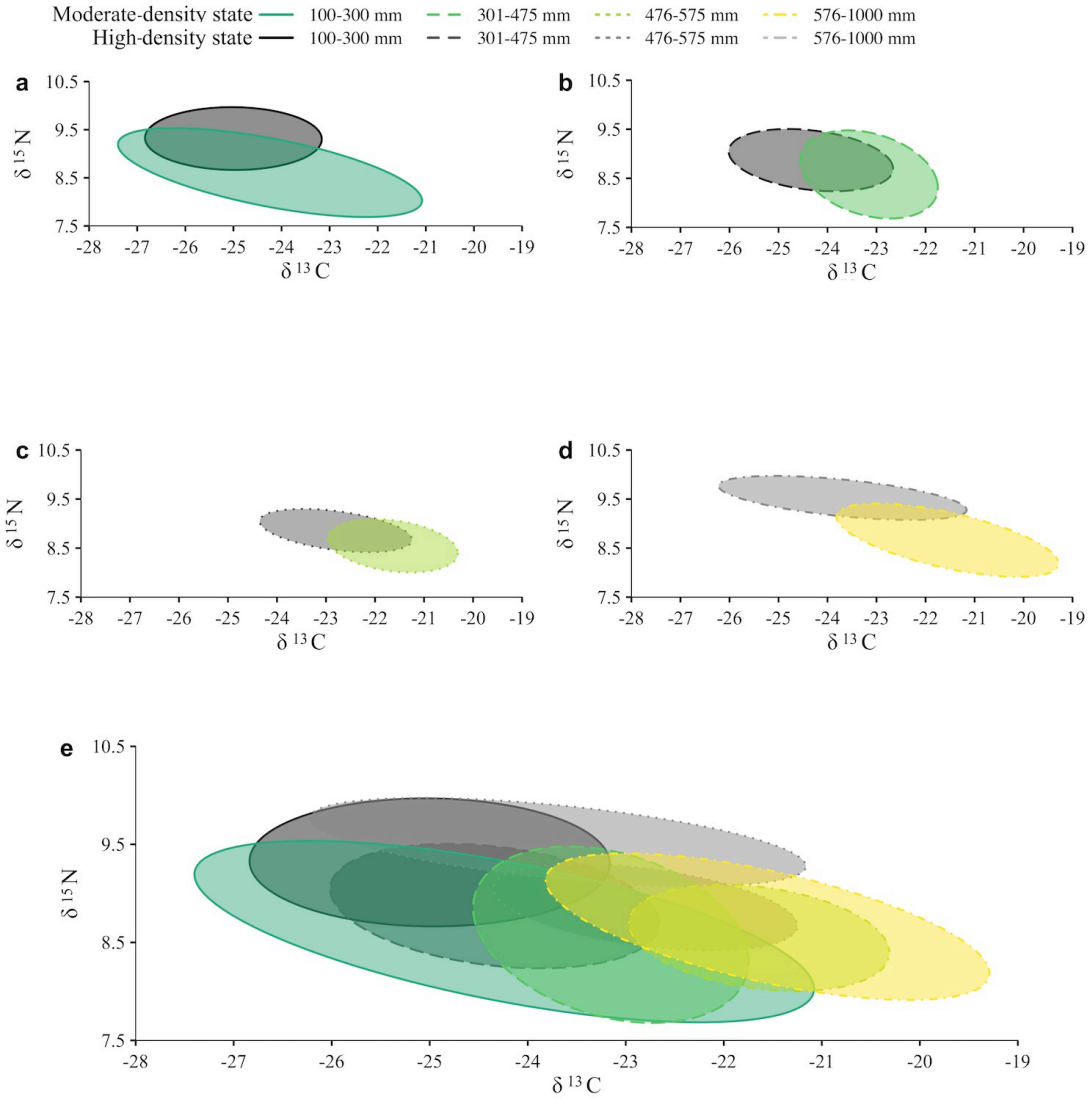
Stable isotope ellipse overlap for lake trout in two lake trout density states. Standard Bayesian ellipse area 40% core distribution of stable isotope signatures for lake trout from a lake trout moderate-density state (green hues) and a lake trout high-density state (Syslo et al. [53]; grey hues) among length classes: 100–300 mm (a), 301–475 mm (b), 476–575 mm (c), 576–1000 mm (d), and all length classes (e). Relative abundances of lake trout are from Syslo et al. [55], where high density is ~450,000 >300 mm lake trout, and moderate-density state is ~300,000 >300 mm lake trout.

When comparing isotopic overlap between the cutthroat trout and lake trout, isotopic ellipses only overlapped during the high-density state (Fig 6a & 6b). Lake trout were enriched in δ^15^N compared to cutthroat trout for both high- and moderate-density states (Fig 6a & 6b). All isotopic ellipses overlapped between the high- and moderate-density states, though the magnitude of overlap differed between species and among length classes. For cutthroat trout, a high degree of overlap (>60%) existed between the high- and moderate-density state for the 100–300 mm length class (86.7% overlap; Fig 7a & 7d) and the 301–475 mm length class (100% overlap; Fig 7b & 7d). Only 18.1% of the ellipse area overlapped between states for cutthroat trout in the 476–575 mm length class (Fig 7c & 7d). Isotopic ellipses for lake trout overlapped between high- and moderate-density states, though the amount of overlap was <60%. Ellipse overlap between states was 58.2% for the 100–300 mm length class (Fig 8a & 8e), 53.1% for the 301–475 mm length class (Fig 8b & 8e), 47.6% for the 476–575 mm length class (Fig 8c & 8e), and 19.8% for the 576–1000 mm length class (Fig 8d & 8e).

## Discussion

Diet plasticity is a common strategy used by invasive piscivores to maintain biomass in invaded ecosystems [28, 29]; however, studies on invasive piscivores have not explored how diet plasticity may influence suppression outcomes (i.e., reducing abundance or eliminating the invasive species while increasing abundance or fully recovering native species). Our results indicated that the apex invasive predator, lake trout, exhibited diet plasticity due to suppression and the changing abundance of the preferred native prey, cutthroat trout. Lake trout occupied the position of an apex predator immediately after colonizing Yellowstone Lake [54], then shifted their diet to amphipods resulting in a convergent trophic position with cutthroat trout during peak expansion [53]. Finally, lake trout exhibited trophic plasticity by again consuming cutthroat trout, as observed in this study, as lake trout abundance declined because of suppression efforts. The plasticity we observed in lake trout diets demonstrated that relationships between predators and prey can complicate achieving goals relating to invasive species suppression and native species recovery. Diet plasticity may explain why native species recovery goals [31] are not being achieved despite decreases in invasive species densities [31, 55]; if consumption relationships are not linear, recovery will not be linear.

Cutthroat trout diets changed relative to overall trout density. Cutthroat trout fully shifted their diets to amphipods from a lake trout absent state [46] to a high-density state [53], but did not shift their diets to cladocerans from a high- to a moderate-density state. Therefore, we did not observe more negative δ^13^C signatures associated with more reliance on pelagic diet items such as cladocerans. However, previous research indicated amphipods are a preferred prey item for cutthroat trout [44, 52, 53, 60, 75]. When at carrying capacity, intraspecific competition may prevent cutthroat trout from consuming amphipods at high proportions [44, 46, 52] because cutthroat trout can quickly reduce amphipod population abundance [52, 76, 77] and selectively consume larger-bodied amphipods [52, 76]. The initial shift from cladocerans to amphipods in cutthroat trout diet was probably due to increased amphipod availability after cutthroat trout density declined [44, 48, 52, 53]. Lower density of cutthroat trout, due to lake trout predation, released amphipods from predation [52]. Furthermore, we suggest cutthroat trout influence amphipod abundance more than other fishes in Yellowstone Lake because amphipods comprise <10% of diet proportion by weight for longnose sucker [61] and most Leucisids [62]. Cutthroat trout abundance peaked at 3.5 million individuals (1.2–11.2 million; 95% CI) [31, 78] then declined to ~ 1.7 million individuals (1.2–2.3 million; 95% CI) in 1998, immediately after lake trout were established [54]. During the apex of expansion, lake trout abundance peaked at 992,960 (759,050–1,123,690; 95% CI) individuals in 2012 [55]. The loss of about 1 million cutthroat trout from the ecosystem could explain why more amphipods were observed in cutthroat trout diets even during the highest lake trout density.

We observed plasticity in diet proportion, stable isotope signatures, and diet overlap as a function of predator densities. As hypothesized, large lake trout (>300 mm) shifted their diets to cutthroat trout as the density of lake trout decreased; supporting prior studies showing that lake trout consume prey fishes in proportion to relative prey densities in the environment [79–82]. Isotopic niche overlap, which can provide insight into whether dietary overlap may occur [73, 74], was minimal between lake trout and cutthroat trout during the lake trout high-density state [53] and absent in the moderate-density state (this study), suggesting dietary niche partitioning occurred during the moderate-density state [83].

Lack of observed dietary niche overlap during the moderate-density state between cutthroat trout and lake trout can be largely attributed to the lake trout shift towards piscivory, additionally supported by our Schoener’s index of diet overlap analyses. However, lake trout in the moderate-density state that consumed high proportions of cutthroat trout were not enriched in δ^15^N relative to lake trout in the high-density state that consumed low proportions of cutthroat trout. Declines in δ^15^N for lake trout observed in the medium-density state compared to the high-density state are likely due to nutrient dynamics in Yellowstone Lake as opposed to lake trout diet. First, we observed decreases in δ^15^N signatures for cutthroat trout and lake trout from the high- to medium-density state. Dynamics beyond diet are likely driving δ^15^N signatures because cutthroat trout diets did not change between states, yet a decrease in δ^15^N was observed for cutthroat trout. Atmospheric deposition of N has increased due to anthropogenic emissions driving negative trends in basal δ^15^N [84]. Increased atmospheric N deposition over time could lead to decreased δ^15^N signatures for lake trout and cutthroat trout observed in this study, as supported by lowered amphipod δ^15^N signatures. Although δ^15^N lake trout signatures did not increase with piscivory as expected, the observed increase in δ^13^C signatures for piscivorous lake trout during the moderate-density state corroborates the diet shift toward 100–300 mm cutthroat trout and expected δ^13^C signatures for that prey. Our results mirror other isotopic studies where lake trout often exhibit low degrees of isotopic overlap with other invertivore salmonid species [85, 86]; that is, even when consuming similar diets isotopic overlap between lake trout and cutthroat trout was low [53]. However, lake trout can exhibit high degrees of overlap when compared to piscivorous salmonids [56, 87]; even when a diet shift was observed, isotopic overlap was high for lake trout between high- and moderate-density states. From our decadal comparison of diet and stable isotope similarity and overlap, we observed a clear signal that invasive piscivorous lake trout exhibited diet plasticity as the predator and prey populations responded to 24 years of suppression.

As cutthroat trout density increased in response to lake trout suppression, a higher proportion of cutthroat trout in lake trout diets will have important implications for management. The NPS established recovery benchmarks for cutthroat trout and suppression benchmarks for lake trout in Yellowstone Lake. The primary benchmarks are a catch per unit effort (CPUE) of 40 cutthroat trout in fall assessments (~3.5 million individuals), 100,000 lake trout, and a desired condition to “restore cutthroat trout to pre-lake trout abundance” [31]. Secondary benchmarks are a CPUE of 26 cutthroat trout (~1.7 million individuals; [54]) and a desired condition to “restore cutthroat trout to abundance during early stages of lake trout invasion” [31]. The NPS met the secondary benchmark for cutthroat trout in 2013 (CPUE = 27) and 2017 (CPUE = 26) [31]. Achieving the primary benchmark for lake trout would reestablish the low-density state observed in 1998, and lake trout diet would be likely be composed of an overwhelming majority of cutthroat trout [54], though the size structure of the population may shift towards more abundant, smaller lake trout.

Lake trout consuming cutthroat trout is likely the largest cause of cutthroat trout decline, as each piscivorous lake trout was estimated to consume 41 cutthroat trout per year [54]. During 1996, the estimated lake trout population (≥3 years of age) consumed approximately 522,000 cutthroat trout [54]. Even at low lake trout abundances, cutthroat trout were declining because of predation, whirling disease, and increased frequency of climate change-induced drought conditions [88, 89]. Thus, we present evidence that the primary desired condition to “restore cutthroat trout to pre-lake trout abundance” [31] in the presence of a low-density lake trout population is questionable given the observed lake trout diet plasticity and the continued presence of whirling disease and climate change effects. Nevertheless, suppression of lake trout benefits the Yellowstone Lake ecosystem [31] and revised suppression benchmarks could focus on more realistic desired conditions given the knowledge gained on predator-prey dynamics from the last seven decades [44–47, 53, 54].

Broad niches driven by diet plasticity are considered one of the most important characteristics of invasive species, benefiting colonization, establishment, and spread of the species once introduced [7, 11, 12, 90, 91]. Diet plasticity benefits apex predators by increasing resilience to environmental stochasticity, such as changes in the availability of prey species [92–95]. High trophic plasticity is often attributed to successful expansion and establishment of invasive species. For example, invasive black rats (*Rattus rattus*) exhibit diet plasticity by shifting their diet to sea turtle hatchlings in the absence of seabird prey [11] allowing them to maintain high densities on islands. Broad and plastic isotopic niches have allowed invasive Gobiidae to establish in the Great Lakes [74], while invasive juvenile largemouth bass (*Micropterus salmoides*) displayed diet plasticity, leading to successful establishment in Iberian streams [96]. Only adult lake trout in Yellowstone Lake exhibit trophic plasticity, which could explain why the population has been resilient to suppression. Given the availability of different prey species, lake trout in Yellowstone Lake can successfully partition resources, even during suppression [55], regardless of cutthroat trout density.

**Fig 9:**
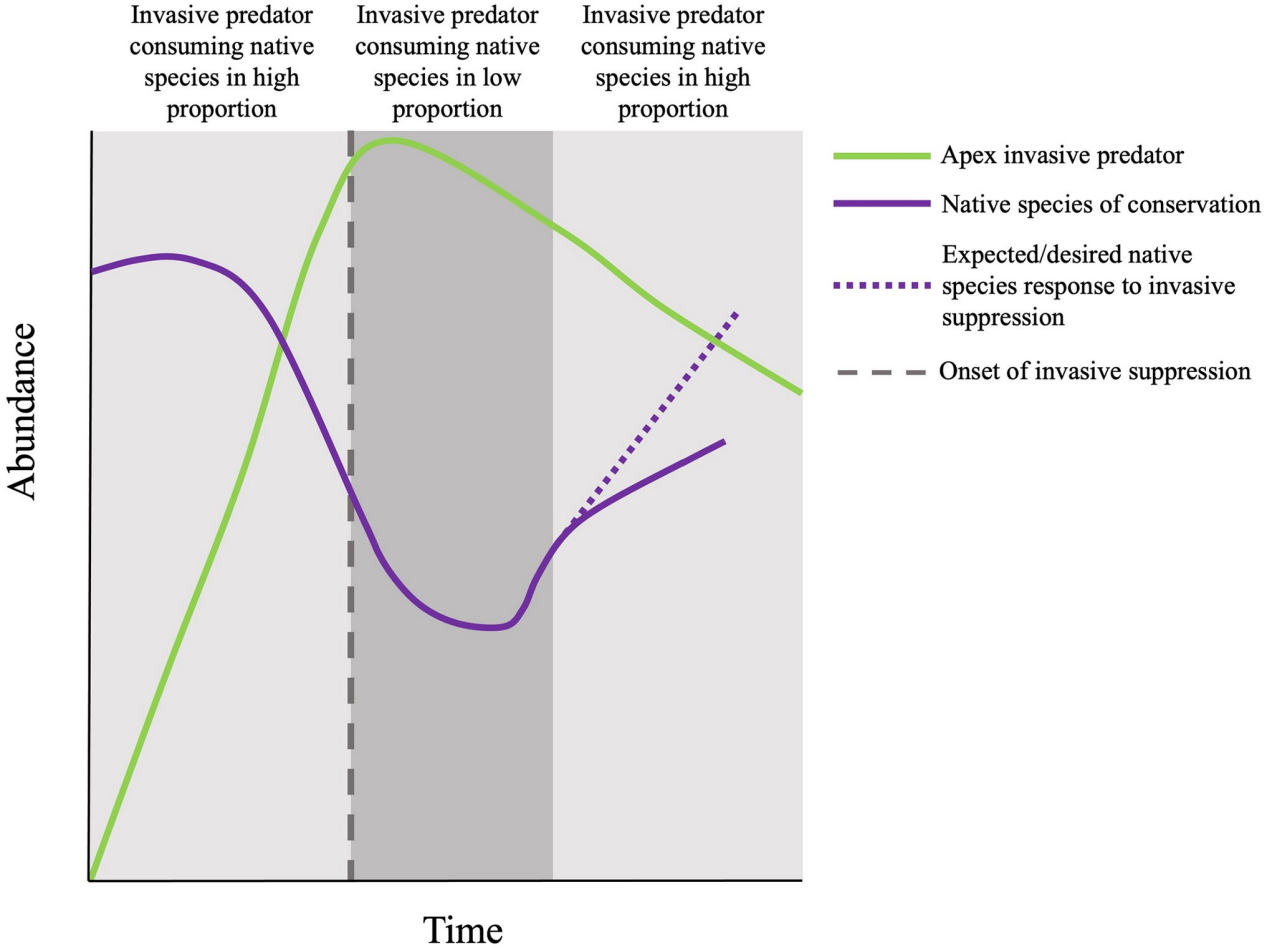
Conceptual model representing dynamics of an apex invasive predator undergoing suppression and a native species of conservation responding to the diet plasticity of the invasive species. Solid lines represent abundances over time from the start of invasive species expansion to the most recent time of suppression. Grey hues represent the diet plasticity of the invasive predator. Light grey polygons represent periods in which the invasive predator consumed their preferred prey, the native species of conservation, in high diet proportion. The dark grey polygon represents a period in which the invasive predator consumed a less-preferred prey in high diet proportion.

Understanding the response of predators to suppression techniques is essential for success. Management programs often focus on predator response by monitoring density, but species removal or suppression can result in unexpected changes to other components of the ecosystem [22], including food-web interactions [11]. Predator diets can be used as a sampling tool, both to monitor prey species abundance (e.g., [97–100]) and changes in invasive predator behavior (e.g., [11, 101]). Identifying dietary plasticity in an invasive predator in response to suppression [101] can aid adaptive management by targeting removal efforts. Eradicating or suppressing an invasive predator can sometimes increase the consumption of native species; therefore, studying food-web interactions can inform management of invasive species [22] (Fig 9).

Our conceptual model of the effects of apex predator diet plasticity and suppression efforts illustrate the complexities and delay in recovery of a native species in need of conservation (Fig 9). For example, during the period of invasive apex predator expansion, the invasive apex predator consumed the preferred prey (species in need of conservation) in high proportion, causing a decline in the preferred prey abundance. Once the invasive predator caused a decline in the preferred prey, the invasive predator switched their diet to the less-preferred prey. The native species in need of conservation begins to recover during the period when the invasive species is consuming the less-preferred prey because severe predation pressure is released (Fig 9). As suppression efforts cause a decline in the invasive apex predator, natural resource agencies would expect the abundance of the native species in need of conservation (Fig 9) to recover to the same abundance observed before invasive species expansion. However, the abundance of the native species in need of conservation either cannot recover or may take longer to recover to the densities observed before invasive expansion because of the diet plasticity of the invasive species; as density of the invasive predator decreases and the native species increases, the invasive predator switches back to consuming the native species in need of conservation (Fig 9).

Our study furthers the understanding of predator-prey dynamics, and how those dynamics influence the success of conservation efforts via invasive predator suppression. We would expect to observe an intersection point in the relationship between cutthroat trout density, lake trout density, and proportion of cutthroat trout in lake trout diets over time. Theoretical predator-prey dynamics [102, 103] predict the relationships we observed among lake trout, cutthroat trout, and amphipods in Yellowstone Lake. When preferred prey density is low, the predator switches to alternative food, can maintain their density, and predation pressure is simultaneously relaxed on preferred prey, thereby allowing the prey population to recover [103]. This theoretical intersection point could provide the key to refining cutthroat trout recovery benchmarks. Lake trout are not likely to be extirpated from Yellowstone Lake; therefore, it is imperative to understand the effects of varying predation pressure (because of prey switching) by lake trout on cutthroat trout abundance and how the varying abundance of cutthroat trout cascades throughout the Yellowstone Lake ecosystem. Concomitantly, revising the conservation benchmarks to better reflect the knowledge of predator-prey dynamics in Yellowstone Lake could provide more realistic benchmarks for the National Park Service.

## Supporting information

S1 Fig Standard Bayesian ellipse area posterior distributions for Yellowstone cutthroat trout for lake trout high-density state (Syslo et al. 2016) and a lake trout moderate-density state (this study) (a), and lake trout for a high lake trout density state (Syslo et al. 2016) and lake trout moderate-density state (this study) (b) among length classes (listed below x-axis; mm total length). Black points represent the median, and boxes present the 50%, 80%, and 95% credible intervals. Relative abundances of lake trout are from Syslo et al. (2020), where no lake trout is a state with no detectable density or complete absence of lake trout, low-density state is ~80,000 >300 mm lake trout, high-density state is ~450,000 >300 mm lake trout, and moderate-density state is ~300,000 >300 mm lake trout.
(DOCX)
