# Supplementary material for: Invasive predator diet plasticity has implications for native fish conservation and invasive species suppression
Source: PLoS One. 2023 Feb 24;18(2):e0279099. doi: 10.1371/journal.pone.0279099 (PMC9956068; doi:10.1371/journal.pone.0279099)
Supplement: S1 Fig — Standard Bayesian ellipse area posterior distributions for Yellowstone cutthroat trout for lake trout high-density state (Syslo et al. 2016) and a lake trout moderate-density state (this study) (a), and lake trout for a high lake trout density state (Syslo et al. 2016) and lake trout moderate-density state (this study) (b) among length classes (listed below x-axis; mm total length). Black points represent the median, and boxes present the 50%, 80%, and 95% credible intervals. Relative abundances of lake trout are from Syslo et al. (2020), where no lake trout is a state with no detectable density or complete absence of lake trout, low-density state is ~80,000 >300 mm lake trout, high-density state is ~450,000 >300 mm lake trout, and moderate-density state is ~300,000 >300 mm lake trout. (DOCX) [file pone.0279099.s001.docx]

Full title: Invasive predator diet plasticity has implications for native fish conservation & invasive species suppression

Short title: Invasive predator diet plasticity has implications for native fish conservation & invasive species suppression

Author: Hayley C. Glassic^1,2^

[hglassic@usgs.gov](mailto:hglassic@usgs.gov); <https://orcid.org/0000-0001-6839-1026>

Contributions: Secured funding, conceived the study, performed the analyses, interpreted results, and wrote the manuscript.

Co-Author: Christopher S. Guy^3^

Contributions: Secured funding, conceived the study, discussed results and implications, and edited earlier manuscripts.

Co-Author: Dominique R. Lujan^4^

Contributions: Discussed results and implications and edited earlier manuscripts.

Co-Author: Lusha M. Tronstad^5^

Contributions: Discussed results and implications and edited earlier manuscripts.

Co-Author: Michelle A. Briggs^2^

Contributions: Discussed results and implications and edited earlier manuscripts.

Co-Author: Lindsey K. Albertson^6^

Contributions: Discussed results and implications and edited earlier manuscripts.

Co-Author: Todd M. Koel^7^

Contributions: Secured funding, discussed results and implications, and edited earlier manuscripts.

^1^ current affiliation: U.S. Geological Survey, Northern Rocky Mountain Science Center, Bozeman, MT, 59717, USA

^2^ Montana Cooperative Fishery Research Unit, Department of Ecology, Montana State University, Bozeman, MT, 59717, USA

^3^ U.S. Geological Survey, Montana Cooperative Fishery Research Unit, Department of Ecology, Montana State University, Bozeman, MT, 59717, USA

^4^ Department of Zoology and Physiology, University of Wyoming, Laramie, WY 82071 USA

^5^ Wyoming Natural Diversity Database, University of Wyoming, Laramie, WY 82071, USA

^6^ Department of Ecology, Montana State University, Bozeman, MT 59717, USA

^7^ U.S. National Park Service, Yellowstone Center for Resources, Native Fish Conservation Program, Yellowstone National Park, WY, 82190, USA

**Data availability statement:** Accession numbers and/or DOIs will be made available after acceptance.

# Supplemental figure

#
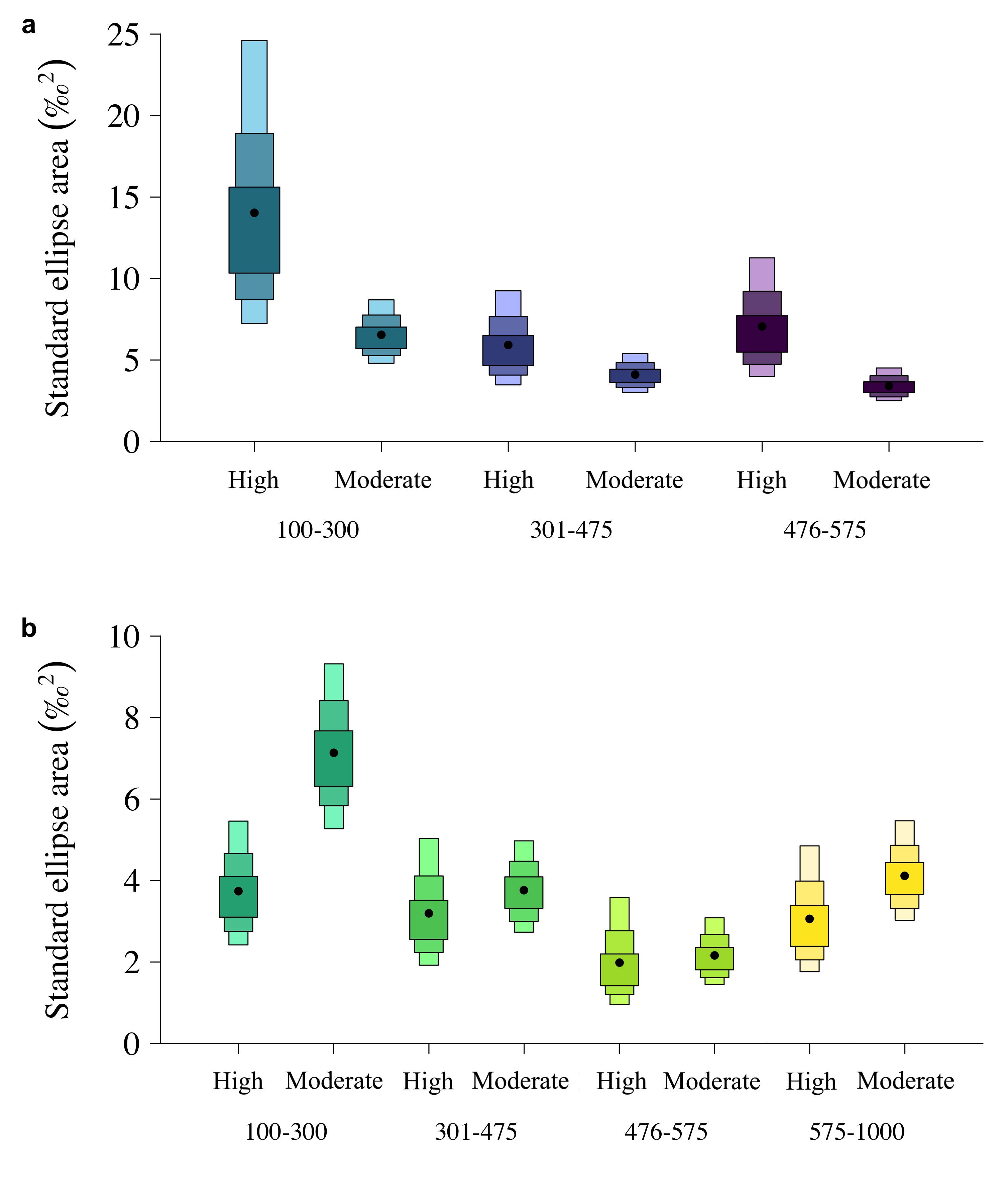


# Fig. S1. Standard Bayesian ellipse area (SEA_B_) posterior distributions for Yellowstone cutthroat trout for lake trout high-density state (Syslo et al. 2016) and a lake trout moderate-density state (this study) (a), and lake trout for a high lake trout density state (Syslo et al. 2016) and lake trout moderate-density state (this study) (b) among length classes (listed below x-axis; mm total length). Black points represent the median, and boxes present the 50%, 80%, and 95% credible intervals. Relative abundances of lake trout are from Syslo et al. (2020), where no lake trout is a state with no detectable density or complete absence of lake trout, low-density state is ~80,000 >300 mm lake trout, high-density state is ~450,000 >300 mm lake trout, and moderate-density state is ~300,000 >300 mm lake trout.
